# Non-contrast-enhanced MR-angiography of the abdominal arteries: intraindividual comparison between relaxation-enhanced angiography without contrast and triggering (REACT) and 4D contrast-enhanced MR-angiography

**DOI:** 10.1007/s00261-024-04639-4

**Published:** 2024-10-28

**Authors:** Carsten Gietzen, Jan Paul Janssen, Lukas Görtz, Kenan Kaya, Thorsten Gietzen, Roman Johannes Gertz, Henry Pennig, Katharina Seuthe, David Maintz, Philip S. Rauen, Thorsten Persigehl, Kilian Weiss, Lenhard Pennig

**Affiliations:** 1 Faculty of Medicine and University Hospital Cologne, Institute for Diagnostic and Interventional Radiology, University of Cologne, Kerpener Straße 62, 50937 Cologne, Germany; 2 Department of Cardiology, Faculty of Medicine and University Hospital Cologne, Heart Center, University of Cologne, Cologne, Germany; 3 Philips GmbH, Hamburg, Germany; 4 Department of Orthopedics and Trauma Surgery, University Hospital Bonn, Bonn, Germany

**Keywords:** Abdominal vessel imaging, Compressed SENSE, Visceral arteries, Contrast-enhanced magnetic resonance angiography, Relaxation-enhanced angiography without contrast and triggering

## Abstract

**Purpose:**

To evaluate Relaxation-Enhanced Angiography without Contrast and Triggering (REACT), a novel 3D isotropic flow-independent non-contrast-enhanced magnetic resonance angiography (non-CE-MRA) for imaging of the abdominal arteries, by comparing image quality and assessment of vessel stenosis intraindidually with 4D CE-MRA.

**Methods:**

Thirty patients (mean age 35.7 ± 16.8 years; 20 females) referred for the assessment of the arterial abdominal vasculature at 3 T were included in this retrospective, single-centre study. The protocol comprised both 4D CE-MRA and REACT (navigator-triggering, Compressed SENSE factor 10, nominal scan time 02:54 min, and reconstructed voxel size 0.78 × 0.78 × 0.85  mm^3^). Two radiologists independently evaluated 14 abdominal artery segments for stenoses, anatomical variants, and vascular findings (aortic dissection, abdominal aorta aneurysms and its branches). Subjective image quality was assessed using a 4-point Likert scale (1 = non-diagnostic, 4 = excellent).

**Results:**

REACT had a total acquisition time of 5:36 ± 00:40 min, while 4D CE-MRA showed a total acquisition time (including the native scan and bolus tracking sequence) of 3:45 ± 00:59 min (p = 0.001). Considering 4D CE-MRA as the reference standard, REACT achieved a sensitivity of 87.5% and specificity of 100.0% for relevant (≥ 50%) stenosis while detecting 89.5% of all vascular findings other than stenosis. For all vessels combined, subjective vessel quality was slightly higher in 4D CE-MRA (3.0 [IQR: 2.0; 4.0.]; P = 0.040), although comparable to REACT (3.0 [IQR: 2.0; 3.5]).

**Conclusion:**

In a short scan time of about 5 min, REACT provides good diagnostic performance for detection of relevant stenoses, variants, and vascular findings of the abdominal arteries, while yielding to 4D CE-MRA comparable image quality.

## Introduction

Abdominal vascular imaging can be performed using a number of different techniques, including computed tomography angiography (CTA) [1], magnetic resonance angiography (MRA) [2, 3], contrast-enhanced ultrasound [4], and digital subtraction angiography (DSA) [5]. For MRA, contrast-enhanced magnetic resonance angiography (CE-MRA) is regarded as the clinical reference standard for the assessment of visceral artery anatomy and patency [6, 7]. CE-MRA can be performed using either first-pass or time-resolved (4D) approaches. However, CE-MRA is limited by the need for intravenous contrast administration, which has associated drawbacks, including the potential for nephrogenic systemic fibrosis in end-stage renal disease [8], uncertain long-term effects of gadolinium deposition [9], allergic reactions [10], and potential mistiming of image acquisition [11].

Therefore, various non-CE-MRA techniques have been developed for the imaging of visceral arteries [12], of which balanced steady-state free precession (bSSFP) [13] and quiescent-interval slice-selective (QISS) MRA [14, 15] have shown promising results and are currently the most widely used techniques. Recently, the Relaxation-Enhanced Angiography without Contrast and Triggering (REACT) sequence has been introduced as a novel non-CE-MRA technique [16]. The REACT sequence permits the simultaneous delineation of arteries and veins, and has demonstrated encouraging results in imaging the thoracic vasculature [17–20], extracranial arteries [21–23], and pelvic vessels [24]. Nevertheless, its performance regarding the depiction of visceral arteries remains to be established.

The aim of this study was to evaluate the REACT sequence for the imaging of the visceral arteries by comparing the detection of visceral artery stenoses, variants and other pathologies as well as image quality with 4D CE-MRA in patients referred for abdominal vascular imaging at 3 T.

## Methods

Approval for this single-centre study was granted by the local institutional review board. In light of the retrospective nature of the study, the institutional review board waived the requirement for written informed consent (reference number: blinded for submission).

### Patient population

The authors reviewed the institutional image database at a tertiary care university hospital for visceral MRA studies between January 2021 and July 2023. Patients were included in the study if they had undergone a standardized protocol for the assessment of the arterial abdominal vasculature at 3 T, which included both REACT and 4D CE-MRA. The exclusion criteria were the absence or technical failure of any of the MRA sequences.

The following data were obtained from the medical records or observed during magnetic resonance imaging (MRI): age, sex, body mass index, indication for MRA, risk factors for atherosclerosis, and ascites.

### Magnetic resonance imaging

A commercially available 3 T MRI system (Philips Ingenia, Philips Healthcare, Best, The Netherlands) with a standard 28-channel body coil without additional modifications was utilized. The abdominal scan protocol comprised T2-weighted turbo spin echo sequences in both the coronal and axial planes, REACT, 4D CE-MRA, and T1-weighted contrast-enhanced gradient-echo sequences with mDIXON for fat suppression in coronal and axial orientations.

For non-CE-MRA, 3D isotropic flow-independent REACT was acquired in the coronal plane covering the entire abdominal aorta from the diaphragm to the common iliac arteries. The REACT sequence comprises of T2 preparation and IR prepulses (which enhance the native blood signal with long T1 and T2) and a water and fat selective Dixon reconstruction based on a 7-peak fat model (mDIXON XD, Philips Healthcare), resulting in suppressed signal from adjacent background and fat [16, 25]. To account for respiratory motion, diaphragmatic pencil beam navigation was employed. The navigator was positioned on the dome of the right hemidiaphragm, and a 6 mm gating window was employed during end-expiration. Furthermore, the coronal image acquisition was combined with sagittal excitation (ENCASE, Philips Healthcare) to enable a reduced field of view in the left–right direction and to mitigate the impact of moiré artifacts [26]. Immediate image reconstruction was conducted using the standard hardware provided by the manufacturer. Compressed SENSE [27] (Philips Healthcare), which combines compressed sensing [28] and parallel imaging using SENSitivity Encoding (SENSE) [29], was used for acceleration of image acquisition. A variable-density incoherent sampling pattern was employed, whereby high-density sampling was conducted in the k-space centre and continuously increasing undersampling was applied towards the k-space periphery for the purpose of data acquisition. The data consistency and image sparsity were ensured by means of iterative L1 norm minimization for image reconstruction. Furthermore, the reconstruction was regularized through the use of SENSE parallel imaging and coil sensitivity distribution. An acceleration factor of 10 was employed, resulting in a nominal scan time of 2 min and 54 s.

For 4D CE-MRA, a 3D spoiled gradient echo sequence was employed. First, a native image was acquired as a mask. Afterwards, Gadobutrol (Gadovist, Bayer HealthCare Pharmaceuticals, Berlin, Germany; 0.1 ml/kg body weight)) was administered into an antecubital vein at a flow rate of 2 mL/second, followed by a 30 mL saline flush. Without triggering, acquisition in coronal plane was initiated after arrival of the contrast agent in the abdominal aorta, as determined by a bolus tracking sequence. Patients were instructed to maintain an end-expiratory breath hold during data acquisition. To achieve high spatiotemporal resolution, the acquisition was combined with SENSE (factor 6) and a keyhole technique with 20% of the central k-space data acquired in each dynamic (4D TRACK, Philips Healthcare) [30], resulting in a nominal scan time of 00:54 min. During reconstruction, the keyhole data were combined with outer k-space data from a reference scan.

Table 1 provides an overview of the imaging parameters of the MRA sequences.

**Table 1:** Imaging parameters of *REACT* relaxation-enhanced angiography without contrast and triggering and *4D CE-MRA* contrast-enhanced magnetic resonance angiography

MRA technique	REACT	4D CE-MRA
Acquisition orientation	Coronal	Coronal
K-space trajectory	Cartesian	Cartesian
Field of view (FH x RL x AP)	400 × 300 × 319 mm^3^	400 × 400 × 140 mm^3^
Acquired voxel size	1.7 × 1.7 × 1.7 mm^3^	0.8 × 1.35 × 2.8 mm^3^
Reconstructed voxel size	0.78 × 0.78 × 0.85 mm^3^	0.620 × 0.620 × 1.4 mm^3^
Flip angle	15°	25°
Repetition time (TR)	6.0 ms	3.8 ms
Echo time (TE, 1/2)	1.32/2.5 ms	1.08 ms
T2 preparation	50 ms; refocusing pulses 4	n/a
IR delay	100 ms	n/a
Temporal resolution	n/a	1 s
Total aquisition time	5:36 min	3:45 min
Subtraction	n/a	CE-native
Image reconstruction	15 s	1 min
Accleration factor	Compressed SENSE 10	SENSE 6

*FH* foot head, *RL* right left, *AP* anterior posterior, *SENSE* sensitivity encoding

### Image analysis

Two readers with three (R1) and six (R2) years of experience in MRA used a commercially available image viewer (DeepUnity Diagnost 1.1.1.1; Dedalus Healthcare Group, Bonn, Germany) to conduct independent reviews of the MRA images during separate sessions and in a randomized order. The readers were free to modify the window level and blinded to clinical and patient data. A four-week interval was maintained between the evaluation of the REACT and 4D CE-MRA datasets to minimize the potential for recall bias.

#### Assessment of subjectiv image quality

Based on vessel delineation, signal intensity, and contrast to adjacent tissue, the readers rated the vessel quality of the MRA datasets using a 4-point Likert scale (1: non-diagnostic, 2: poor, 3: fair, 4: excellent). The following arterial vessels were analyzed:

1. Suprarenal abdominal aorta (SRA)
2. Infrarenal abdominal aorta (IRA)
3. Celiac trunk (CT)
4. Superior mesenteric artery (SMA)
5. Splenic artery (SA)
6. Right renal artery (RRA)
7. Left renal artery (LRA)
8. Common hepatic artery (CHA)
9. Proper hepatic artery (PHA)
10. Gastroduodenal artery (GDA)
11. Left gastric artery (LGA)
12. Right hepatic artery (RHA)
13. Left hepatic artery (LHA)
14. Inferior mesenteric artery (IMA)

#### Assessment of visceral artery patency

In order to perform a subjective assessment of visceral vessel stenosis, MRA datasets were graded for stenosis affecting any of the aforementioned vessels using a 1–5 grading scale: Grade 1: regular patency, grade 2: stenosis, < 50% of vessel lumen, grade 3: stenosis, 50%-69% of vessel lumen, grade 4: stenosis, ≥ 70–99% of vessel lumen, grade 5: vessel occlusion. In the event of multiple stenoses, the most severe lesion was deemed the diagnostic grade and subjected to further analysis.

#### Assessment of vascular variants and other vascular findings

Furthermore, readers were advised to evaluate potential anatomical variants of the visceral arteries, including variants of hepatic arterial anatomy, aberrant renal arteries, and direct origin of the visceral arteries from the abdominal aorta. Moreover, readers were directed to assess MRAs for additional vascular findings beyond stenosis including vascular dissection and aneurysms of the aortic branches or visceral arteries.

### Statistical analysis

The statistical analysis was conducted using IBM SPSS Statistics software (version 25.0, Armonk, NY, USA). The Shapiro–Wilk test was employed to ascertain a normal distribution. Categorical variables are presented as frequencies and corresponding percentages. Quantitative variables are presented as mean and standard deviation. Subjective ratings are presented as the median and interquartile range. Comparisons of normally distributed quantitative data were conducted using the Student's t-test, while non-normally distributed ordinal scaled data were compared using the Wilcoxon signed-rank test. A p-value of less than 0.05 was considered statistically significant for two-tailed tests.

Sensitivity and specificity of REACT regarding the detection of stenoses and other vascular findings were calculated considering 4D CE-MRA as a reference standard [7]. Cohen's kappa was employed to evaluate interrater and intersequence concordance in the identification of stenosis and other vascular findings. The interpretation of the degree of agreement was as follows: 0.01–0.2 slight, 0.21–0.4 fair, 0.41–0.6 moderate, 0.61–0.8 substantial, and 0.81–0.99 almost perfect.

## Results

### Study population and baseline characteristics

**Fig. 1:**
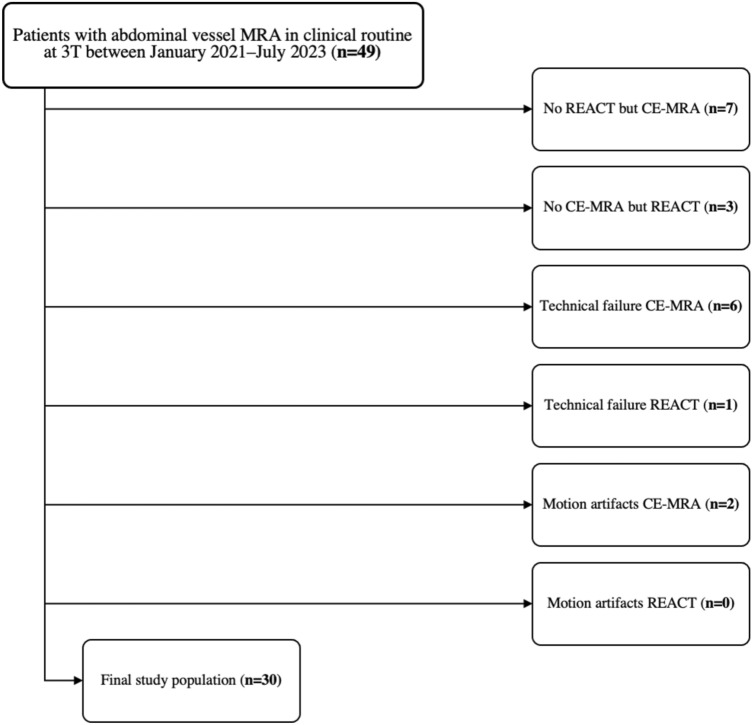
Workflow for patient inclusion and exclusion. *MRA* magnetic resonance angiography, *T* Tesla, *4D CE-MRA* time-resolved contrast-enhanced magnetic resonance angiography, *REACT* relaxation-enhanced angiography without contrast and triggering

A total of 49 consecutive patients underwent imaging of the visceral arteries with 4D CE-MRA and REACT between January 2021 and July 2023. Seven patients were excluded from the study due to the absence of REACT, three patients due to the absence of 4D CE-MRA. Six patients were excluded due to technical failure of the 4D CE-MRA, one patient was excluded due to technical failure of the REACT. Due to motion artifacts two patients had to be excluded in 4D CE-MRA, no patient in REACT. Therefore, the final study population consisted of 30 patients (mean age 35.7 ± 16.8 years; 20 females). Each reader evaluated 60 datasets (30 datasets each for REACT and 4D CE-MRA), resulting in a total number of 840 visceral artery segments. Figure 1 illustrates the process for applying inclusion and exclusion criteria. Table 2 presents an overview of patient characteristics and cardiovascular risk factors.

**Table 2:** Patient characteristics; MRA = magnetic resonance angiography, SD = standard deviation

Patients, n	30	
Age (years, mean ± SD)	35.7 ± 16.8	
BMI (mean ± SD)	28.1 ± 11.2	
	n	%
Gender		
Female	20	66.6
Male	10	33.3
Indication for MRA		
Renal artery stenosis	20	66.6
Aortic dissection	4	13.3
Aortic aneurysm	6	20.0
Cardiovascular risk factors		
Arterial hypertension	25	83.3
Diabetes mellitus	0	0.0
Dyslipidemia	5	16.6
Smoking	1	3.3
Ascites	5	16.6

### Acquisition time

The total acquisition time of REACT including image reconstruction, was 05:36 ± 00:40 min, whereas 4D CE-MRA (including the native scan and bolus tracking sequence) requiered 03:45 ± 00:59 min (p < 0.001).

### Assessment of subjective image quality

For all vessels combined, subjective vessel quality was slightly higher in 4D CE-MRA (median 3.0 [IQR: 2.0; 4.0.]; P < 0.040), although comparable to REACT (3.0 [IQR: 2.0; 3.5]). While both MRA sequences showed similar results for the abdominal aorta, 4D CE-MRA provided higher values for the majority of the aortic branches, including CT (4.0 [IQR: 3.5; 4.0] vs. 3.5 [IQR: 3.5; 3.6]; P = 0.011), SMA (4.0 [IQR: 3.5; 4.0] vs. 3.5 [IQR: 3.5; 4.0]; P = 0.003), and RRA (3.5 [IQR: 3.0; 4.0] vs. 3.3 [IQR: 3.0; 3.5]; P = 0.006). Regarding medium-sized arteries, 4D CE-MRA yielded higher scores at the CHA (3.5 [IQR: 2.9; 4.0]; P = 0.033), compared to REACT (3.0 [IQR: 2.5; 3.5]), whereas the remaining arteries of this size and small-sized arteries showed no difference. Table 3 provides detailed results of image quality assessment for all vessel segments pooled for both readers.

**Table 3:** Vessel image quality scores of REACT and 4D CE-MRA, pooled for both readers, bold indicates statistical significance

	4D CE-MRA		REACT		P
	Median (IQR)	Mean	Median (IQR)	Mean	
Overall, all arteries	3 (2–4)	3.0 ± 0.9	3 (2–3.5)	2.8 ± 0.9	**0.040**
Aorta					
Aorta, suprarenal	4 (4–4)	4.0 ± 0.2	4 (4–4)	3.9 ± 0.4	0.160
Aorta, infrarenal	4 (4–4)	3.9 ± 0.2	4 (4–4)	3.9 ± 0.4	0.527
Large aortic branches					
CT	4 (3.5–4)	3.8 ± 0.3	3.5 (3.5–3.6)	3.5 ± 0.5	**0.011**
SMA	4 (3.5–4)	3.9 ± 0.3	3.5 (3.5–4)	3.6 ± 0.5	**0.003**
RRA	3.5 (3–4)	3.5 ± 0.5	3.25 (3–3.5)	3.2 ± 0.5	**0.006**
LRA	3.5 (3–4)	3.4 ± 0.6	3.5 (3 -3.5)	3.2 ± 0.6	0.276
Medium sized arteries					
SA	3 (2.9–3.5)	3.1 ± 0.8	3.25 (2.5–3.5)	3.0 ± 0.6	0.418
CHA	3.5 (2.9–4)	3.2 ± 0.7	3.0 (2.5–3.5)	2.9 ± 0.6	**0.033**
PHA	2.5 (1.9–3)	2.5 ± 0.8	2.5 (2–3)	2.4 ± 0.7	0.298
Small sized arteries					
LGA	2 (1.5–2.5)	2.2 ± 0.6	2 (1.5–2.5)	2.1 ± 0.7	0.494
GDA	2 (1.9–2.5)	2.2 ± 0.6	2 (1.5–2.5)	2.1 ± 0.6	0.454
LHA	1.75 (1.5–2)	1.9 ± 0.6	1.75 (1.5–2.1)	1.9 ± 0.5	1.0
RHA	2 (1.5–2.5)	2.0 ± 0.7	1.75 (1.5–2.1)	1.9 ± 0.6	0.283
IMA	2.5 (2–3)	2.6 ± 0.6	2.5 (2–2.5)	2.4 ± 0.5	0.110

Scoring scales of 1–5 were used, 1 being non-diagnostic

*REACT* relaxation-enhanced angiography without contrast and triggering, *4D CE-MRA* contrast-enhanced magnetic resonance angiography, *CT* celiac trunk, *SMA* superior mesenteric artery, *RRA* right renal artery, *LRA* left renal artery, *SA* splenic artery, *CHA* common hepatic artery, *PHA* proper hepatic artery, *LGA* left gastric artery, *GDA* gastroduodenal artery, *LHA* left hepatic artery, *RHA* right hepatic artery, *IMA* inferior mesenteric artery, *IQR* interquartile range, *SD* standard deviation

#### Assessment of visceral artery patency

In 4D CE-MRA, a total of 15 visceral artery stenoses were found in 30 patients. Eight of these were considered clinically relevant. Using 4D CE-MRA as the reference standard, REACT provided a sensitivity of 70.0% and specificity of 99.0% for any and a sensitivity of 87.5% and specificity of 100.0% for clinically relevant visceral artery stenosis. Regarding stenosis grading, REACT obtained a substantial intersequence agreement with 4D CE-MRA for any (Cohen's Kappa 0.65) and an almost perfect agreement with 4D CE-MRA for relevant stenosis (Cohen's Kappa 0.89). Interobserver agreement was comparable between both MRA sequences, with substantial agreement for REACT (Kendall´s W 0.78) and almost perfect agreement for 4D CE-MRA (Kendall´s W 0.85), as demonstrated in Table 4.

**Table 4:** Interobserver agreement for stenosis grading in 4D contrast-enhanced magnetic resonance angiography (CE-MRA) and *REACT* relaxation-enhanced angiography without contrast and triggering assessed by Kendall's W (0.01–0.2 slight, 0.21–0.4 fair, 0.41–0.6 moderate, 0.61–0.8 substantial, and 0.81–0.99 almost perfect)

Interobserver agreement	REACT	4D CE-MRA
Grading overall	0.778	0.850
All stenosis	0.800	0.771
Relevant stenosis	0.756	0.929

#### Assessment of vascular variants and other vascular findings

**Fig. 2:**
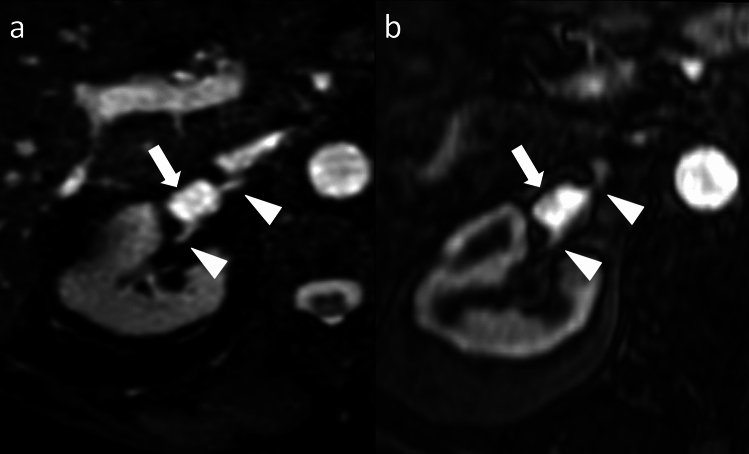
Axial source images of relaxation-enhanced angiography without contrast and triggering (REACT, **a** and 4D contrast-enhanced magnetic resonance angiography (4D CE-MRA, **b** depicting the right renal artery in a 42-year-old male patient with suspected renal artery stenosis due to resistant arterial hypertension. The renal artery aneurysm (arrow) can be delineated in both MRA techniques while REACT shows an improved delineation of the vessel wall of both aneurysm and parent artery (arrowheads) compared to a blurry appearance in 4D CE-MRA

**Fig. 3:**
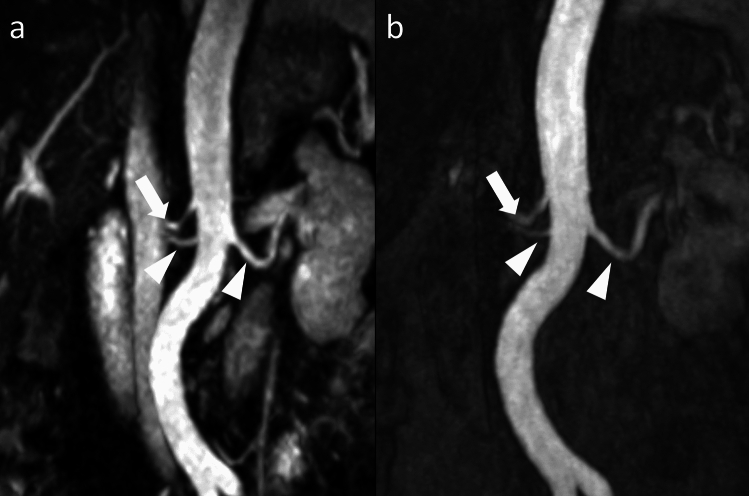
Maximum intensity projections (MIP, slice thickness 15 mm) in coronal reformation of Relaxation-Enhanced Angiography without Contrast and Triggering (REACT, **a** and 4D contrast-enhanced magnetic resonance angiography (4D-CE-MRA, **b** in a 23-year-old male patient with Marfan syndrome referred for abdominal aortic assessment. Both sequences enable the exclusion of stenosis of the renal arteries (arrowheads) while depicting a right-sided aberrant upper renal artery (arrows)

**Fig. 4:**
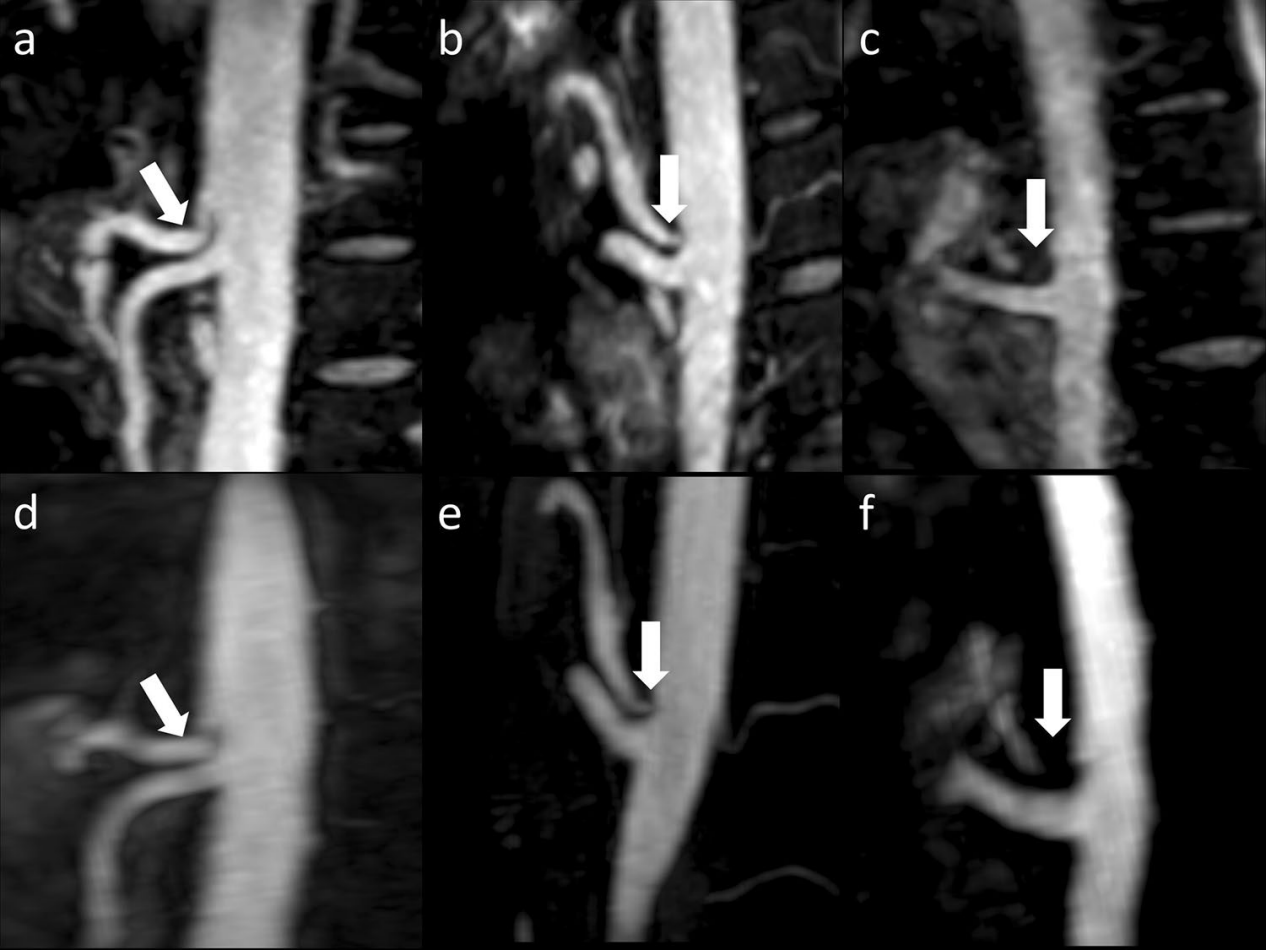
Maximum intensity projections (MIP, slice thickness: 10 mm) in sagittal reformation and angulation to the celiac trunk (arrow) in Relaxation-Enhanced Angiography without Contrast and Triggering (REACT; **a**, **b**, **c**) and 4D contrast-enhanced magnetic resonance angiography (4D CE-MRA; **d**, **e**, **f**) in three patients refered for suspected visceral artery stenosis. REACT depicts a low (**a**, **d**; 33-year-old female patient), moderate (**b**, **e**; 42-year-old male patient), and high grade stenosis (**c**, **g**; 50-year-old male patient) of the celiac trunk in good intersequence agreement to 4D CE-MRA while yielding a superior vessel delination given motion artifacts in 4D-CE-MRA

**Fig. 5:**
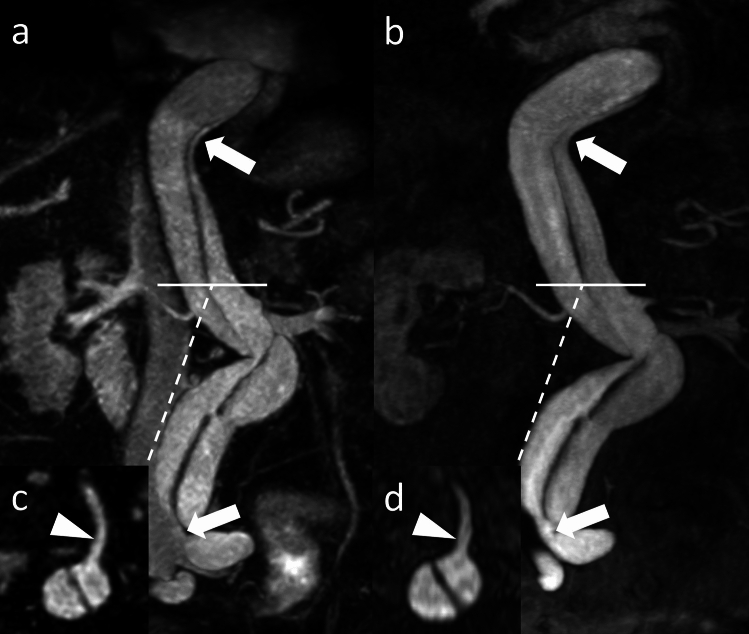
Maximum intensity projections (MIP, slice thickness: 10 mm) in coronal reformation in Relaxation-Enhanced Angiography without Contrast and Triggering (REACT, **a**) and 4D contrast-enhanced magnetic resonance angiography (4D CE-MRA, **b**) in a 75-year-old male patient referred for follow up after abdominal aortic dissection. Both REACT and 4D CE-MRA enable a precise delineation of the dissection affecting the abdominal aorta and the ilical bifurcation (arrows) with source images in axial reformation of REACT (**c**) and 4D CE-MRA (**d**) at the level of the celiac trunk depicting the origin of the celiac trunk arising from the true lumen (arrowheads)

Considering 4D CE-MRA as the reference standard, REACT yielded a sensitivity of 87.5% and specificity of 100.0% for the delineation of vascular variants while providing a sensitivity of 100.0% and specificity for the detection of other vascular findings (dissection and aneurysms). Combined, REACT obtained a sensitivity of 89.5% and specificity of 100.0% for all vascular findings other than stenosis. Super­numerary renal arteries were the most common vascular variants followed by variant hepatic arterial anatomy. Other vascular findings included abdominal aortic dissection in one patient and aneurysms of the renal and spenic arteries in two and one patient, respectively. A detailed summary of vascular variants and other vascular findings is given in Table 5. REACT yielded an excellent intersequence agreement with 4D CE-MRA for the detection of vascular variants and other findings (Cohen's Kappa 0.89) while the interobserver agreement was excellent in both REACT (Cohen's Kappa 0.97) and 4D CE-MRA (Cohen's Kappa 0.88). Figures 2, 3, 4, and 5 give exemplary comparisons of REACT-non-CE-MRA and 4D CE-MRA.

**Table 5:** Vascular anomalies and vascular findings for both readers’ combined, decimal numbers were rounded to the nearest whole number

Vascular anomalies	REACT (n, %)	4D CE-MRA (n, %)	Sensitivity REACT (%)	Specificity REACT (%)
Aberrant renal artery	7/30 (23.3%)	8/30 (26.7%)	87.5%	100.0%
Aberrant hepatic artery	2/30 (6.6%)	4/30 (13.3%)	50.0%	100.0%
Aberrant gastric artery	1/30 (1.6%)	1/30 (3.3%)	100.0%	100.0%
Aberrant splenic artery	3/30 (10.0%)	3/30 (10.0%)	100%	100.0%
Vascular findings				
Abdominal aortic dissection	1/30 (3.33%)	1/30 (3.33%)	100%	100.0%
Renal artery aneurysm	2/30 (6.6%)	2/30 (6.6%)	100%	100.0%
Splenic artery aneurym	1/30 (3.3%)	1/30 (3.3%)	100%	100.0%
Overall	17/30	19/30	89.5%	100.0%

## Discussion

In this work, a novel flow-independent non-contrast MRA technique (REACT) was evaluated for imaging the visceral arteries at 3 T by comparing subjective image quality and assessment of visceral artery stenosis, variants as well as other vascular findings with 4D CE-MRA in patients suspected for visceral artery stenosis. The main findings of this study are the following:

1. REACT provides a good diagnostic performance for the detection of clinically relevant visceral artery stenoses, variants, and other findings.

2. Without gadolinium-based contrast agents, REACT provided to 4D CE-MRA comparable image quality of the visceral arteries in a short scan time of about 5 min.

Compared to 4D CE-MRA, REACT obtained a sensitivity of 70% and specificity of 99% for all and a sensitivity of 87.5% and specificity of 100% for clinically relevant (≥ 50%) stenosis of the visceral arteries. Additionally, REACT yielded a substantial interobserver agreement (Kendall’s W 0.756) and an almost perfect intersequence agreement for grading of disease (Cohen's kappa 0.89). These findings are consistent with previously published non-CE-MRA studies for the visceral arteries which mainly focused on renal and pelvic artery patency. For the assessment of clinically relevant renal artery stenosis using free-breathing inflow IR-bSSFP-MRA compared to CE-MRA, Glockner et al. reported sensitivities of 82–94% and specificities of 82–86% at 1.5 T [31] whereas Lal et al. reported 97–98% and 95–98% at 3 T. The results of the latter study demonstrated near-perfect agreement between IR-bSSFP-MRA and CE-MRA regarding grading of disease (Cohen's kappa 0.9–0.93). For quadruple IR-bSSFP-MRA at 1.5 T compared to 4D CE-MRA, Atanasova et al. reported a sensitivity of 86% and a specificity of 93–94% for clinically relevant stenosis of the renal and iliac arteries. Additionally, the study demonstrated a substantial interobserver agreement (Cohen's kappa 0.77) [32]. Vargas-Szemes et al. investigated free-breathing QISS-MRA for the depiction of the abdominal aorta and pelvic arteries with the fast low-angle shot-(FLASH) based QISS-MRA approach yielding the best results with a sensitivity of 84% and a specificity of 94% compared to CTA for clinically relevant stenosis of pelvic vessels with substantial interobserver agreement (Cohen's kappa 0.721) [14]. Regarding the detection of vascular findings other than stenosis, e.g. aneurysms or aberrant arteries, REACT showed a sensitivity of 89.5% and a specifity of 100%. These findings are in line with quadruple IR-bSSFP-MRA for which Atanasova et al. reported a sensitivity of 75–100% [32] regading the delineation of accessory renal arteries and renal artery aneurysms. Similarly, for IR-bSSFP-MRA Glockner et al. reported a sensitivity of 93% and specificity of 100% for the detection of aberrant renal arteries [31]. For QISS-MRA, Adnersson et al. reported a sensitivity of 86% and a specificity of 100% for super-numerary renal arteries in patients prior to living kidney donation [33].

In accordance with prior research investigating bSSFP-MRA for visceral artery imaging [31, 32], REACT had to 4D CE-MRA comparable but slightly inferior image quality. These findings are mainly due to the inherent limitations of non-CE-MRA with long acquisition time leading to subsequent bowel motion and corresponding artifacts [34]. Furthermore, REACT yields a lower in-plane resolution compared to 4D CE-MRA and most importantly provides a simultaneous visualization of arteries and veins [16, 17]. While being beneficial for the assessment of large directly adjacent aortic and venous thoracic vessels in congential heart disease in a single scan [17–20], it poses a limitation of this sequence technique when vessels are yielding a smaller size, leading to potential overshadowing and limited assessment. In this context, image quality at the aorta was comparable between both techniques, while REACT yielded an inferior image quality at the aortic branches. Interestingly, the RRA showed poorer results compared to the LRA in REACT, most likely due to the adjacent inferior vena cava affecting the assessment of the RRA. Of note, small sized arteries did not yield significantly different image quality, potentially due to breathing artifacts in 4D CE-MRA counterbalancing the above referenced limitations of REACT which is acquired during end-expiratory respiratory state using navigator-triggering. In future, we are aiming to replace the iterative reconstruction algorithm of REACT by adding deep learning-based image reconstruction in order to improve the spatial resolution, which has already shown encouraging results in musculoskeletal imaging [35].

4D CE-MRA with high temporal resolution allows for the acquisition of a series of volume angiograms in rapid succession, thereby simplifying the timing of acquisition in relation to the passage of the contrast bolus [34]. This is of particular interest in abdominal imaging to subsequently depict arterial, portal venous, and venous vessels in a single scan [36]. Nevertheless, the precise execution of 4D CE-MRA is technically demanding, as image quality is contingent upon optimal coordination between contrast injection, precise timing of data acquisition, and patient cooperation during breath-holding. This restricts the applicability of these techniques in noncompliant patients [12–14, 37, 38].

In contrast, non-CE-MRA techniques such as bSSFP, can be acquired during free breathing using respiratory gating or navigator echo tracking [12] with the acquisition to be repeated as often as required. Modifications of bSSFP using arterial spin labeling [39], inflow-based techniques [31], IR prepulses [31, 32], and ECG-synchronisation [40] have improved image quality over time. ECG-gated QISS-MRA can be acquired during multiple breath-holds using the bSSFP readout or during free-breathing employing the FLASH technique [14]. Nevertheless, the resulting long acquisition times to cover the whole visceral vasculature in bSSFP-MRA (6 min) [31] and QISS-MRA (7 min) [14] restrain their feasibility in clinical routine with motion artifacts from peristaltic motion and nonuniform respiratory displacements, being inherent limitations of non-CE-MRA [34]. Furthermore, the necessity for ECG-synchronization of both bSSFP and QISS-MRA to obtain high image quality represents an additional limitation, given that abdominal imaging is generally performed without cardiac or pulse triggering. Moreover, the requirement for ECG synchronization of both bSSFP and QISS-MRA represents an additional challenge, as abdominal imaging is typically conducted without cardiac or pulse triggering [31, 32]. In contrast, REACT is acquired without ECG-synchronisation [16], which facilitates its use in clinical routine. The application of Compressed SENSE allows for the reduction of the scan time to a duration of about five minutes. Additionally, REACT exploits the specific relaxation properties of blood and is independent of blood flow, whereas QISS-MRA relies on the inflow of spins from outside the saturation volume, leading to blood flow dependency and 2D anisotropic image volumes with low through-plane resolution (up to 3 mm) [14]. On the contrary, REACT provides high isotropic resolution (0.78 × 0.78 × 0.85 mm^3^), which is comparable to bSSFP-MRA (0.7 × 0.7 × 1.0 mm^3^) [41] with both sequences enabling multiplanar reformation of the abdominal vasculature.

### Limitations

We acknowledge that our retrospective single-centre study has several limitations. Firstly, there was no comparison of REACT with CTA or DSA with the latter being the reference standard for visceral artery imaging. Secondly, it was not possible to blind the readers to the type of MRA due to different appearances of 4D CE-MRA and REACT, which could have affected the results. Thirdly, the order of acquisition sequences was not randomized, which may prove disadvantageous for 4D CE-MRA in patients exhibiting motion artifacts towards the conclusion of the examination. Fourthly, image quality was rated exclusively on subjective evaluation, lacking objective criteria such as signal-to-noise ratio (SNR). However, the disparate acceleration techniques of the MRA sequences with Compressed SENSE of REACT (using wavelet denoising resulting in apparent SNR, which may vary from image to image because of the different number of denoising steps resulting in potential recurring errors of quantitative SNR measurements) and 4D CE-MRA (parallel imaging), preclude a comprehensive comparison of SNR and CNR. Fifthly, no comparison of REACT and 3D CE-MRA was performed in this study. 3D CE-MRA would enable a higher spatial resolution compared to 4D CE-MRA and potentially more accurate in vascular assessment but at the expense of missing dynamic information and increased requirements on sequence timing [42]. However, future work comparing 3D CE-MRA with REACT may nurture further investigation. Sixthly, since the REACT sequence enables a simultaneous depiction of arteries and veins [16] the resulting signals of the venous vasculature may be regarded as a limitation potentially leading to a crowded image with decreased diagnostic performance. In this context, the adjustment of the T2 preparation pulse in REACT may enhance the difference in contrast between arteries and veins, by the divergent oxygenation of venous and arterial blood. Finally, the selected Compressed SENSE factor for REACT was based on the authors' clinical experience, without a direct comparison of different undersampling factors. It is conceivable that even higher acceleration factors, preferably combined with deep learning-based image reconstructions, may be feasible. This could result in a reduction of scan time while improving spatial resolution, which would be a valuable contribution to future research on REACT.

## Conclusion

In a short scan time of about five minutes, Compressed SENSE accelerated REACT offers satisfactory diagnostic capabilities for identifying relevant artery stenosis, anatomical variants, and other abdominal artery findings, while simultaneously providing image quality comparable to 4D CE-MRA. These findings highlight the potential of REACT for imaging the abdominal vasculature without the use of gadolinium contrast agents.

## Data Availability

The datasets generated and/or analyzed during the current study are not publicly available due to data protection but are available from the corresponding author upon reasonable request. The imaging protocol of the proposed REACT sequence is available from the corresponding author upon reasonable request.

## Abbreviations

CE-MRA: Contrast-enhanced magnetic resonance angiography
CHA: Common hepatic artery
CT: Celiac trunk
GDA: Gastroduodenal artery
IMA: Inferior mesenteric artery
IQR: Interquartile range
LGA: Left gastric artery
LHA: Left hepatic artery
LRA: Left renal artery
MIP: Maximum intensity projection
PHA: Proper hepatic artery
QISS: Quiescent interval slice-selective
REACT: Relaxation-enhanced angiography without contrast and triggering
RHA: Right hepatic artery
RRA: Right renal artery
SA: Splenic artery
SD: Standard deviation
SMA: Superior mesenteric artery
SRA: Suprarenal aorta
IRA: Infrarenal aorta
SENSE: Sensitivity encoding
TOF: Time-of-flight

## References

[1] Lennartz S, Laukamp KR, Tandon Y, et al (2021) Abdominal vessel depiction on virtual triphasic spectral detector CT: initial clinical experience. Abdom Radiol (NY) 46:3501–3511. 10.1007/s00261-021-03001-2 33715050 10.1007/s00261-021-03001-2 PMC8215039

[2] Ekelund L, Sjöqvist L, Thuomas KA, Asberg B (1996) MR angiography of abdominal and peripheral arteries. Techniques and clinical applications. Acta Radiol 37:3–13. 10.1177/02841851960371P103 8611320 10.1177/02841851960371P103

[3] Korosec FR, Frayne R, Grist TM, Mistretta CA (1996) Time-resolved contrast-enhanced 3D MR angiography. Magn Reson Med 36:345–351. 10.1002/mrm.1910360304 8875403 10.1002/mrm.1910360304

[4] Pfister K, Kasprzak PM, Jung EM, et al (2016) Contrast-enhanced ultrasound to evaluate organ microvascularization after operative versus endovascular treatment of visceral artery aneurysms. Clin Hemorheol Microcirc 64:689–698. 10.3233/CH-168003 27802212 10.3233/CH-168003

[5] Huber TS, Björck M, Chandra A, et al (2021) Chronic mesenteric ischemia: Clinical practice guidelines from the Society for Vascular Surgery. J Vasc Surg 73:87S-115S. 10.1016/j.jvs.2020.10.029 33171195 10.1016/j.jvs.2020.10.029

[6] Liu X, Zhang W, Li Z, et al (2019) Improved display of abdominal contrast-enhanced MRA using gadobutrol: comparison with Gd-DTPA. Clin Radiol 74:978.e1-978.e7. 10.1016/j.crad.2019.08.012 31551147 10.1016/j.crad.2019.08.012

[7] Leiner T, Ho KY, Thelissen GR, et al (1999) [Contrast-enhanced magnetic resonance angiography]. Ned Tijdschr Geneeskd 143:1087–93 10368744

[8] Perazella MA (2009) Advanced kidney disease, gadolinium and nephrogenic systemic fibrosis: the perfect storm. Curr Opin Nephrol Hypertens 18:519–525. 10.1097/MNH.0b013e3283309660 19623065 10.1097/MNH.0b013e3283309660

[9] Gulani V, Calamante F, Shellock FG, et al (2017) Gadolinium deposition in the brain: summary of evidence and recommendations. Lancet Neurol 16:564–570. 10.1016/S1474-4422(17)30158-8 28653648 10.1016/S1474-4422(17)30158-8

[10] Jung J-W, Kang H-R, Kim M-H, et al (2012) Immediate Hypersensitivity Reaction to Gadolinium-based MR Contrast Media. Radiology 264:414–422. 10.1148/radiol.12112025 22550309 10.1148/radiol.12112025

[11] Menke J (2009) Carotid MR angiography with traditional bolus timing: clinical observations and Fourier-based modelling of contrast kinetics. Eur Radiol 19:2654–2662. 10.1007/s00330-009-1448-9 19449013 10.1007/s00330-009-1448-9 PMC2762047

[12] Miyazaki M, Isoda H (2011) Non-contrast-enhanced MR angiography of the abdomen. Eur J Radiol 80:9–23. 10.1016/j.ejrad.2011.01.093 21330081 10.1016/j.ejrad.2011.01.093

[13] Schieda N, Isupov I, Chung A, et al (2017) Practical applications of balanced steady-state free-precession (bSSFP) imaging in the abdomen and pelvis. Journal of Magnetic Resonance Imaging 45:11–20. 10.1002/jmri.25336 27373694 10.1002/jmri.25336

[14] Varga-Szemes A, Aherne EA, Schoepf UJ, et al (2019) Free-Breathing Fast Low-Angle Shot Quiescent-Interval Slice-Selective Magnetic Resonance Angiography for Improved Detection of Vascular Stenoses in the Pelvis and Abdomen. Invest Radiol 54:752–756. 10.1097/RLI.0000000000000592 31299678 10.1097/RLI.0000000000000592 PMC6832800

[15] Edelman RR, Sheehan JJ, Dunkle E, et al (2010) Quiescent-interval single-shot unenhanced magnetic resonance angiography of peripheral vascular disease: Technical considerations and clinical feasibility. Magn Reson Med 63:951–958. 10.1002/mrm.22287 20373396 10.1002/mrm.22287 PMC2896273

[16] Yoneyama M, Zhang S, Hu HH, et al (2019) Free-breathing non-contrast-enhanced flow-independent MR angiography using magnetization-prepared 3D non-balanced dual-echo Dixon method: A feasibility study at 3 Tesla. Magn Reson Imaging 63:137–146. 10.1016/j.mri.2019.08.017 31425807 10.1016/j.mri.2019.08.017

[17] Pennig L, Wagner A, Weiss K, et al (2020) Imaging of the pulmonary vasculature in congenital heart disease without gadolinium contrast: Intraindividual comparison of a novel Compressed SENSE accelerated 3D modified REACT with 4D contrast-enhanced magnetic resonance angiography. Journal of Cardiovascular Magnetic Resonance 22:8. 10.1186/s12968-019-0591-y 31969137 10.1186/s12968-019-0591-y PMC6977250

[18] Gietzen C, Pennig L, von Stein J, et al (2023) Thoracic aorta diameters in Marfan patients: Intraindividual comparison of 3D modified relaxation-enhanced angiography without contrast and triggering (REACT) with transthoracic echocardiography. Int J Cardiol 390:131203. 10.1016/j.ijcard.2023.131203 37480997 10.1016/j.ijcard.2023.131203

[19] Pennig L, Wagner A, Weiss K, et al (2021) Comparison of a novel Compressed SENSE accelerated 3D modified relaxation-enhanced angiography without contrast and triggering with CE-MRA in imaging of the thoracic aorta. Int J Cardiovasc Imaging 37:315–329. 10.1007/s10554-020-01979-2 32852711 10.1007/s10554-020-01979-2 PMC7878228

[20] Betz LH, Dillman JR, Towbin AJ, et al (2023) Respiratory-Triggered Flow-Independent Noncontrast Non–ECG-Gated MRV (REACT) Versus CE-MRV for Central Venous Evaluation in Children and Young Adults: A Six-Reader Study. American Journal of Roentgenology 221:240–248. 10.2214/AJR.22.28893 36946900 10.2214/AJR.22.28893

[21] Hoyer UCI, Lennartz S, Abdullayev N, et al (2022) Imaging of the extracranial internal carotid artery in acute ischemic stroke: assessment of stenosis, plaques, and image quality using relaxation-enhanced angiography without contrast and triggering (REACT). Quant Imaging Med Surg 12:3640–3654. 10.21037/QIMS-21-1122/COIF) 35782261 10.21037/qims-21-1122 PMC9246733

[22] Pennig L, Kabbasch C, Hoyer UCI, et al (2021) Relaxation-Enhanced Angiography Without Contrast and Triggering (REACT) for Fast Imaging of Extracranial Arteries in Acute Ischemic Stroke at 3 T. Clin Neuroradiol 31:815–826. 10.1007/s00062-020-00963-6 33026511 10.1007/s00062-020-00963-6 PMC8463375

[23] Gietzen C, Kaya K, Janssen JP, et al (2024) Highly compressed SENSE accelerated relaxation-enhanced angiography without contrast and triggering (REACT) for fast non-contrast enhanced magnetic resonance angiography of the neck: Clinical evaluation in patients with acute ischemic stroke at 3 tesla. Magn Reson Imaging. 10.1016/j.mri.2024.04.009 38599503 10.1016/j.mri.2024.04.009

[24] Terwolbeck MN, Zhang S, Bode M, et al (2021) Relaxation-Enhanced Angiography without Contrast and Triggering (REACT) for pelvic MR venography in comparison to balanced gradient-echo and T2-weighted spin-echo techniques. Clin Imaging 74:149–155. 10.1016/j.clinimag.2020.12.029 33607595 10.1016/j.clinimag.2020.12.029

[25] Eggers H, Brendel B, Duijndam A, Herigault G (2011) Dual-echo Dixon imaging with flexible choice of echo times. Magn Reson Med 65:96–107. 10.1002/MRM.22578 20860006 10.1002/mrm.22578

[26] Stadler A, Schima W, Ba-Ssalamah A, et al (2007) Artifacts in body MR imaging: their appearance and how to eliminate them. Eur Radiol 17:1242–55. 10.1007/s00330-006-0470-4 17149625 10.1007/s00330-006-0470-4

[27] Liang D, Liu B, Wang J, Ying L (2009) Accelerating SENSE using compressed sensing. Magn Reson Med 62:1574–1584. 10.1002/mrm.22161 19785017 10.1002/mrm.22161

[28] Lustig M, Donoho D, Pauly JM (2007) Sparse MRI: The application of compressed sensing for rapid MR imaging. Magn Reson Med 58:1182–1195. 10.1002/mrm.21391 17969013 10.1002/mrm.21391

[29] Pruessmann KP, Weiger M, Scheidegger MB, Boesiger P (1999) SENSE: sensitivity encoding for fast MRI. Magn Reson Med 42:952–62 10542355

[30] Willinek WA, Hadizadeh DR, von Falkenhausen M, et al (2008) 4D time‐resolved MR angiography with keyhole (4D‐TRAK): More than 60 times accelerated MRA using a combination of CENTRA, keyhole, and SENSE at 3.0T. Journal of Magnetic Resonance Imaging 27:1455–1460. 10.1002/jmri.21354 18504736 10.1002/jmri.21354

[31] Glockner JF, Takahashi N, Kawashima A, et al (2010) Non-contrast renal artery MRA using an inflow inversion recovery steady state free precession technique (Inhance): comparison with 3D contrast-enhanced MRA. J Magn Reson Imaging 31:1411–8. 10.1002/jmri.22194 20512894 10.1002/jmri.22194

[32] Atanasova IP, Lim RP, Chandarana H, et al (2014) Quadruple inversion-recovery b-SSFP MRA of the abdomen: initial clinical validation. Eur J Radiol 83:1612–9. 10.1016/j.ejrad.2014.05.026 24998363 10.1016/j.ejrad.2014.05.026 PMC4706232

[33] Andersson J, Meik R, Pravdivtseva M, et al (2024) Preoperatively determining renal perfusion and visualizing renal arteries and any abnormalities in potential living kidney donors using non-contrast-enhanced magnetic resonance imaging techniques at 1.5 Tesla. Clin Kidney J. 10.1093/ckj/sfae101 38915436 10.1093/ckj/sfae101 PMC11194483

[34] Roditi G, Wieben O, Prince MR, Hecht EM (2022) MR Angiography Series: Abdominal and Pelvic MR Angiography. Radiographics 42:E94–E95. 10.1148/rg.210224 35245106 10.1148/rg.210224

[35] Terzis R, Dratsch T, Hahnfeldt R, et al (2024) Five-minute knee MRI: An AI-based super resolution reconstruction approach for compressed sensing. A validation study on healthy volunteers. Eur J Radiol 175:111418. 10.1016/j.ejrad.2024.111418 38490130 10.1016/j.ejrad.2024.111418

[36] Maj E, Cieszanowski A, Rowiński O, et al (2010) Time-resolved contrast-enhanced MR angiography: Value of hemodynamic information in the assessment of vascular diseases. Pol J Radiol 75:52–60 22802762 PMC3389854

[37] Miyazaki M, Lee VS (2008) Nonenhanced MR Angiography. Radiology 248:20–43. 10.1148/radiol.2481071497 18566168 10.1148/radiol.2481071497

[38] Edelman RR, Koktzoglou I (2019) Non-Contrast MR Angiography:An Update HHS Public Access. J Magn Reson Imaging 49:355–373. 10.1002/jmri.26288 30566270 10.1002/jmri.26288 PMC6330154

[39] Spuentrup E, Manning WJ, Börnert P, et al (2002) Renal arteries: navigator-gated balanced fast field-echo projection MR angiography with aortic spin labeling: initial experience. Radiology 225:589–96. 10.1148/radiol.2252011366 12409599 10.1148/radiol.2252011366

[40] Wyttenbach R, Braghetti A, Wyss M, et al (2007) Renal artery assessment with nonenhanced steady-state free precession versus contrast-enhanced MR angiography. Radiology 245:186–95. 10.1148/radiol.2443061769 17717326 10.1148/radiol.2443061769

[41] Bultman EM, Klaers J, Johnson KM, et al (2014) Non-contrast enhanced 3D SSFP MRA of the renal allograft vasculature: a comparison between radial linear combination and Cartesian inflow-weighted acquisitions. Magn Reson Imaging 32:190–5. 10.1016/j.mri.2013.10.004 24246390 10.1016/j.mri.2013.10.004 PMC3893796

[42] Vogt FM, Theysohn JM, Michna D, et al (2013) Contrast-enhanced time-resolved 4D MRA of congenital heart and vessel anomalies: image quality and diagnostic value compared with 3D MRA. Eur Radiol 23:2392–2404. 10.1007/s00330-013-2845-7 23645330 10.1007/s00330-013-2845-7

